# The Preliminary Development of an *in vitro* Poultry Cecal Culture Model to Evaluate the Effects of Original XPC^TM^ for the Reduction of *Campylobacter jejuni* and Its Potential Effects on the Microbiota

**DOI:** 10.3389/fmicb.2019.03062

**Published:** 2020-01-23

**Authors:** Kristina M. Feye, Peter M. Rubinelli, William Evan Chaney, Hilary O. Pavlidis, Michael H. Kogut, Steven C. Ricke

**Affiliations:** ^1^Southern Plains Agricultural Research Center, Agricultural Research Service, United States Department of Agriculture, College Station, TX, United States; ^2^Department of Food Science, Center for Food Safety, University of Arkansas, Fayetteville, AR, United States; ^3^Diamond V, Cedar Rapids, IA, United States

**Keywords:** *Campylobacter*, microbiota, XPC, competitive exclusion hypothesis, yeast fermentate

## Abstract

Poultry is a major reservoir for the pathogen *Campylobacter jejuni*. *C. jejuni* inhabits the poultry gastrointestinal tract as a part of the gut microbiota. The objective of this study was to evaluate both the survival of *C. jejuni* and the changes in the population dynamics of the cecal microbiome during an *in vitro C. jejuni* inoculation in the presence or absence of the functional metabolites of Diamond V Original XPC^TM^ (XPC). Two independent trials were conducted. Broiler chickens (*n* = 6 per Trial 1 and *n* = 3 per Trial 2) were raised according to standard industry guidelines and euthanized on Day 41. The ceca were collected aseptically, their contents removed independently and then used in an *in vitro* microaerobic model with 0.1% cecal contents + *Campylobacter* with or without 1% XPC (w/v). Before the inoculation with a chloramphenicol resistant marker strain of *C. jejuni*, the cecal contents were pre-incubated with XPC at 42°C for 24 h, in a shaking incubator (200 rpm) under microaerobic conditions, then experimentally inoculated with 10^8^/ml of *C. jejuni* into the appropriate treatment groups. At 0 and 24 h for Trial 1, and 48 h for Trial 2, sub-samples of the culture (*n* = 3 ceca, two technical replicates per ceca, XPC alone or ceca culture alone) were enumerated using a Petroff–Hausser counter, and the DNA was extracted for microbiome analysis. DNA was isolated using the Qiagen QIAamp Fast Stool DNA Mini Kit and sequenced using the Illumina MiSeq platform. The reads were filtered, normalized, and assigned taxonomical identities using the QIIME2 pipeline. The relative microbiota populations were identified via ANCOM. Altogether, evidence suggests that XPC alters the microbiome, and in turn reduces *Campylobacter* survival.

## Introduction

Arguably, very few foodborne bacterial pathogens exhibit as much metabolic and genomic plasticity and adaptability in the face of environmental stressors as *Campylobacter* spp. As a result, *Campylobacter* are difficult to control and may require multi-modal approaches to successfully reduce the pathogen in commercial poultry production globally. While other food animals are known carriers of *Campylobacter*, specific nuances of avian physiology enable *Campylobacter* colonization to be both successful and clinically undetectable in most cases (Sibanda et al., 2018). As a result, *Campylobacter* are ubiquitous on the farm, with prevalence upward of 100% in many instances (Beery et al., 1988; Stern, 2008; Meade et al., 2009; Sibanda et al., 2018). Human exposure occurs via the fecal oral route along the farm to fork continuum as gastrointestinal contents are often disrupted and expressed during evisceration, contaminating the carcass. Contaminated poultry can result in significant morbidity and mortality in humans worldwide (Kirk et al., 2015).

Unlike some foodborne diseases, such as *Salmonella*, *Campylobacter* infections are known to be associated with several autoimmune sequela, such as Guillain–Barré Syndrome and reactive arthritis (Janssen et al., 2008), with the latter resulting in life-threatening paralysis. Therefore, for many reasons, *Campylobacter* is a significant threat to human health and is an extraordinarily difficult challenge to mitigate in commercial poultry production.

As public scrutiny and government regulations increase, significant innovations in pre-harvest control measures are required to help reduce the risk of *Campylobacter* in commercial poultry production. Historically, the use of in-feed antibiotics has been used in food animal production, with limited effectiveness at reducing *Campylobacter*. While debates as to whether or not the agricultural sector contributes to the world-wide rise in antimicrobial resistance, any measurable increase in publicly described antibiotic resistant reservoirs is concerning. As public pressure increases, the production of natural, antibiotic-free products is increasing.

One approach to controlling *Campylobacter* pre-harvest is by influencing the gut microbiome with various probiotic, prebiotic, enzyme, and fermentate compounds to promote the competitive exclusion of *Campylobacter* and the fortification of the host immunobiology-microbiome axis (Baffoni et al., 2012; Guyard-Nicodème et al., 2016; Schneitz and Hakkinen, 2016). Specifically, yeast fermentates likely promote changes to the microbiome that are theorized to aid in the stability of the host-immunobiology axis (Possemiers et al., 2013; Han et al., 2017). Evidence also suggests that the microbiota dictates both the window of opportunity for *Campylobacter* colonization in poultry, while also aiding in the TH_1_ immune response polarization to control *Campylobacter* colonization (Han et al., 2017). Previous research by our group and others demonstrated that the *Saccharomyces cerevisiae* fermentate Original XPC^TM^ (XPC; Diamond V, Cedar Rapids, IA, United States) inhibits *Salmonella* Typhimurium, both in an *in vitro* anaerobic mixed culture assay and *in vivo* (Feye et al., 2016; Rubinelli et al., 2016; Roto et al., 2017).

Based on the results from studies evaluating the anti-*Salmonella* effects of XPC, it is hypothesized that XPC might also be effective in the control of *Campylobacter*. Furthermore, it is hypothesized that the use of XPC beneficially modulates the microbiome and promotes populations of bacteria that are potentially antagonistic to *Campylobacter jejuni*. In doing so, the use of XPC may result in a multi-modal mechanism to reduce *Campylobacter* fitness in chicken ceca. As an initial step in testing this hypothesis, a study was conducted where XPC was applied in an *in vitro* cecal model inoculated with a marker strain of *C. jejuni*. Those results indicate that inclusion of XPC in this model system can significantly reduce *C. jejuni* survival and results in significant changes to the cecal microbial ecology.

## Materials and Methods

### Ceca Acquisition

Cobb 500 broiler ceca were collected during standard poultry processing at a commercial plant by Diamond V for the study. Animals processed in that commercial plant were done in accordance with standard industry guidelines and ethical practices. Therefore, the University of Arkansas and the Center for Food Safety was outside of the prevue of IACUC as the animal work conducted throughout this study was in accordance with industry guidelines. These birds were not raised for the study, but instead were raised for consumption in accordance with standard industry guidelines and practices.

### Bacterial Cultures

A *C. jejuni* marker strain was graciously donated by Dr. Young-Min Kwon, from the Department of Poultry Science at the University of Arkansas in Fayetteville. This strain of *Campylobacter* substitutes the chloramphenicol exporter gene for the non-essential gene hippicurate biosynthesis, Δ*hipO*, thus conferring chloramphenicol resistance. The ΔhipO strain, henceforth known as *C. jejuni*, was grown for 24 h in Mueller-Hinton broth (MHB; BD, Sparks, MD, United States) and cell density was quantified using a Petroff–Hausser Counter (Hausser Scientific, Horsham, PA, United States). This starter culture containing 10^8^ cells/ml *C. jejuni* was subsequently added to 20 mL Bolton Enrichment Broth (Neogen, Lansing, MI, United States). The Bolton Enrichment Broth contained 20 μg/ml cefoperazone, 50 μg/mL cycloheximide, 20 μg/mL trimethoprim, 20 μg/mL vancomycin, and 5% defibrinated horse blood, which further selected and enriched for *Campylobacter* populations. Aliquots from the Bolton enrichment broth cultures were serially diluted with tryptone salt broth and spread-plated on Cefex agar (Neogen, Lansing, MI, United States) containing 33 μg/mL cefoperazone, 5% defibrinated horse blood, and 10 μg/ml chloramphenicol to empirically verify the inoculum concentration.

### *In vitro Campylobacter* Survival Assay

**FIGURE 1:**
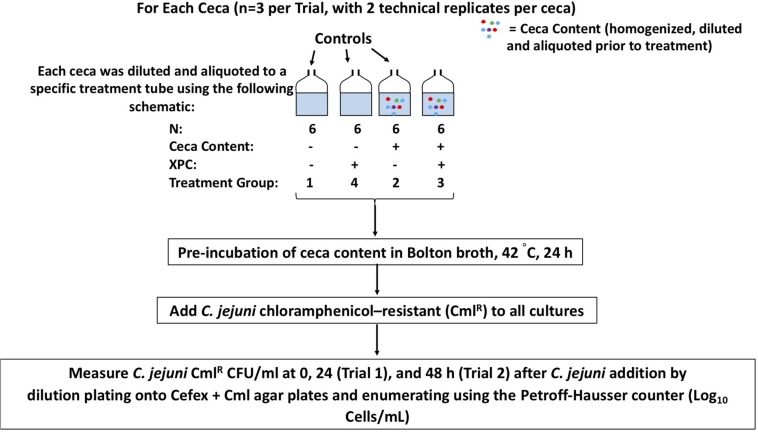
A schematic of the experimental design. Experiment 1 was two independent trials (*n* = 3 ceca each trial, with two technical replicates per cecum), with sample collection at 0 and 24 h. Experiment 2 carried the study out through 48 h.

Two independent trials were conducted throughout the course of this *in vitro* experiment using the ceca collected from commercial broilers by Diamond V. At the processing facility 41-day-old Cobb 500 broiler male ceca were collected, chilled, and shipped overnight from Diamond V (Cedar Rapids, IA, United States). Both trials are delineated in Figure 1. Per treatment in Trial 1, three biological replicates of ceca from three different chickens were used, with two technical replicates executed per ceca and two independent trials conducted in total, resulting in a total of 12 replications per treatment. The aliquots collected at each time point were not sequenced in Trial 1 as specific changes to the microbiota became the focus during the latter half of the study. Per treatment in Trial 2, three ceca were used, with two technical replicates per ceca, with a total of six replicates per treatment. The ceca contents from Trial 2 were sequenced to evaluate the stability of the microbiome after exposure to XPC and *Campylobacter* using our ceca culture model.

As outlined in Figure 1, Trial 1, four treatment groups were tested: (1) *C. jejuni* alone; (2) *C. jejuni* + 0.1% cecal content; (3) *C. jejuni* + cecal + 1% XPC (w/v); (4) *C. jejuni* + 1% XPC (no cecal content). The controls of XPC alone, and *C. jejuni* and XPC alone were added to rule out the direct effects of XPC on *C. jejuni* survival.

In Trial 2, the same treatment groups were followed, except that the following groups were omitted: *C. jejuni* alone and *C. jejuni* + 1% XPC (no cecal content). The adjustment to Trial 2 was made because there were no detectible differences in pathogen load over time in the controls from previous studies (ceca alone, XPC alone). For Trial 2, 0, 24, and 48 h post-inoculation samples were collected.

Irrespective of the trial, the contribution to the ceca with each culture method was standardized, with cecal contents being weighed out to ensure homogeneity between the biological and technical replicates, and then added to 20 mL of Bolton Broth at a final concentration of 0.1% (w/v; ceca to serum bottle volume). Bolton’s Broth was selected as it has been historically used in our laboratory for the long-term maintenance of viable, log-phase *Campylobacter*. Additionally, the antibiotics were included as the background microbiota made *Campylobacter* recovery difficult. Other media were evaluated; however, the successful recovery of *Campylobacter* was not consistent nor accurate using other methods. For each trial, the experimental units were pre-incubated in sterile serum vials with a microaerobic atmosphere (5% O_2_, 10% CO_2_, 85% N_2_) at 42°C, 150 RPM for 24 h (Trial 1) and through 48 h (Trial 2). *C. jejuni* was added to each culture at a final concentration of 1 × 10^8^ cells/mL after the 24 h pre-incubation time (Rubinelli et al., 2016). A 1 mL aliquot was removed from each culture at the start of the experiment and at each time point to count the *C. jejuni* per mL using the Petroff Hausser counter (Ziprin et al., 2003). Petroff-Hausser count data was reported for each trial as it closely paralleled microscopy confirmations of the cell morphology and structure and can be used to detect viable but non-cultivable *Campylobacter* (Ziprin et al., 2003). Because of the difficulty associated with accurately quantifying the *Campylobacter* in microaerobic stasis as identified by pilot studies, using the Petroff-Hausser counter ensured that we also did not report solely prevalence data for *Campylobacter*. As a result, data presented herein is strictly from cell counts.

### Microbiome Sequencing

Throughout Trial 2, DNA was extracted from the aseptically collected aliquots of the microaerobic cultures taken at the indicated time points (0, 24, and 48 h) using the Qiagen Qiamp Fast DNA Stool Mini Kit (Qiagen, Hilden, Germany). The DNA purity was assessed, and then the DNA samples were diluted to 10 ng/mL. The paired-end sequencing libraries were prepared by targeting the hypervariable region 4 of the 16S ribosomal RNA with PCR primers containing the linker and adapter sequence. The libraries were assessed for qualitative and quantitative homogeneity, and then sequenced using the Illumina MiSeq platform as previously described (Kozich et al., 2013).

### Microbiome Bioinformatic Analysis

Data sequences were uploaded onto the BaseSpace Website^1^ (Illumina, San Diego, CA, United States), where sequence run quality and run completion were determined. De-multiplexed data was then downloaded locally and uploaded onto QIIME2-2018.8 via the Casava1.8 paired-end pipeline. All data analysis on QIIME2 were conducted using the q2cli interface. Data were visualized and then trimmed in DADA2 using the chimera consensus pipeline. Alpha and beta diversity were computed via the QIIME phylogeny align-to-tree-mafft-fasttree methodology, then analyzed for all available metrics of alpha and beta diversity via QIIME diversity core-metrics-phylogenetic, with a sampling depth of 14,000 reads for both diversity and alpha-rarefaction analysis. Taxonomic assignment of the operational taxonomical units was conducted using the QIIME feature-classifier classify-sklearn Bayesian methodology with the QIIME2-2018.8 SILVA database. Statistically significant differences in alpha and beta diversity were computed via PERMANOVA. Compositional differential abundance via ANCOM (Analysis of Composition) was conducted and unique phylogenetic features in the relative abundance tables were exported into Microsoft Excel (Microsoft, Redmond, Washington, United States), sorted for the top nine features that were statistically significant (*Q* < 0.05), and visualized.

### Statistical Analysis

The cell counts from the Petroff–Hausser counter were recorded, log_10_ transformed, then inputted into SAS JMP 14.0 (SAS, Cary, NC, United States) where they were evaluated for the main effects of treatment (XPC or CON) and time and the interaction thereof as well as the random effects of experiment. The random effects of trial date were not considered statistically significant. Dunnett corrections for multiple comparisons were implemented *post hoc* to correct for errors in pair-wise comparisons, which were made across all treatment groups relative to the most basic reduced control as identified in the section “Results.” Significance was defined at a *P* ≤ 0.05. The statistical analyses for the microbiome were computed as a component of QIIME2 using standard pipelines. With the microbiome data, when considering alpha and beta diversity as well as the ANCOM analyses, a *P* ≤ 0.05 was considered statistically significant for the overall effect, with *Q* ≤ 0.05 being significant for individual contrasts in order to incorporate a stringent false discovery rate (ANCOM).

## Results

### *Campylobacter* Survival in Cultures in the Presence and Absence of XPC

The effects of XPC has on broilers reqiures the metabolization of the product by the microbiota for its anti-*Salmonella* effects (Rubinelli et al., 2016). *Campylobacter* is a significant foodborne pathogen; therefore, as the poultry industry is constantly trying to evaluate new tools to address this issue, it became important to see if XPC had *in vitro* anti-*Campylobacter* effects. In order to initially evaluate the potential anti-*Campylobacter* effects of XPC, determining whether or not the metabolites produced by XPC *in vitro* reduced *Campylobacter* were essential. Because working with *Campylobacter* is difficult, we modified our traditional *in vitro* model system piloted by Rubinelli et al. (2016) to function for *Campylobacter*. The anerobic model was ineffective; thus, modifications using established media in microaerobic conditions were used after numerous attempts. Antibiotics were included to enhance the recovery of *Campylobacter*. While artificial, it does enable researchers to evaluate trends.

**FIGURE 2:**
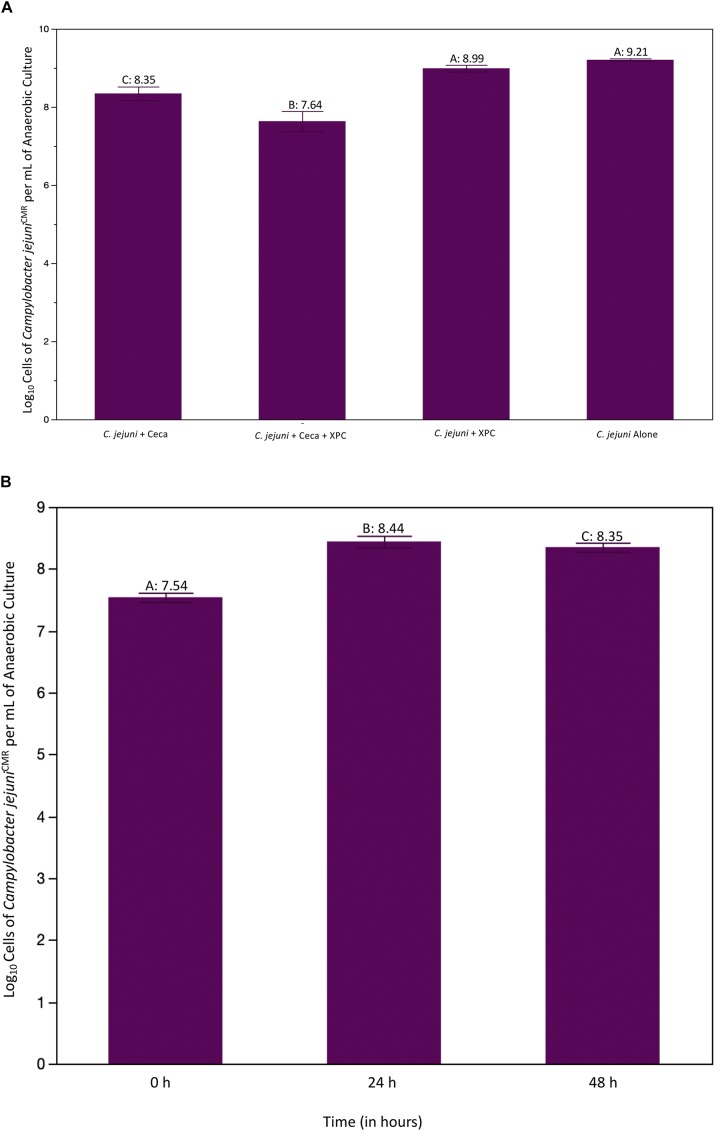
**(A)** Recovered log cells of *Campylobacter jejuni* per mL of ceca culture at 24 h. Ceca were aseptically harvested from 41 day old Cobb 500 male broilers for Trial 1. All comparisons were made against *C. jejuni* in Bolton’s broth only using Dunnet’s Test for multiple comparisons. The bars represent the standard error of the sample mean. Each different letter indicates statistical significance and each number represents the mean Log_10_ CFU recovery of *Campylobacter* (*P* < 0.05). **(B)** Log cells of *C. jejuni* per mL of cecal culture recovered over time. *C. jejuni* were isolate at 0, 24, and 48 h and quantified using a Petroff–Hausser counter. The purple line is the CON group, the aqua line is the XPC treated group. There were significant effects of treatment and time as compared to the CON at 0 h group. There were no differences in XPC and CON at 0 h, therefore all differences are theorized to emerge due to treatment effects.

Irrespective of the trial, 0 h pre-inoculation samples were collected and plated and evaluated with the Petroff–Hausser counter, with no growth observed. The first experiment set out to evaluate whether or not XPC impacted *Campylobacter* directly and whether or not it required the ceca to reduce *C. jejuni* and was repeated two times. The results of Experiment 1 are shown in Figure 2A. All groups were compared to the *C. jejuni* only group using the Dunnet’s test. There was no significant reduction in *C. jejuni* recovered from groups containing XPC alone. However, when XPC was added to cecal contents, there was a 1.57 log reduction in recovered *C. jejuni* (*P* < 0.0001). That reduction was greater the cecal content alone, where the *C. jejuni* recovered from the cecal contents only demonstrated a 0.86 log reduction as compared to the *C. jejuni* group alone (*P* < 0.001). There was no difference between *C. jejuni* + XPC and *C. jejuni* alone throughout the trial, meaning cecal contents are required in order for XPC to produce anti-*C. jejuni* effects. This requirement coincides with observations made in previous studies with a similar cecal *in vitro* system and *Salmonella* (Rubinelli et al., 2016). Comparing the treatments with *C. jejuni* alone, a significant decrease in log-phase *C. jejuni* was observed in the 24 h mixed cultures with XPC compared to 24 h mixed cultures without XPC (Figure 2A).

Trial 2 was conducted to evaluate whether or not the reduction in *C. jejuni* was sustained over time, and if there were substantial shifts in the microbiome. The 48 h time point was added to the trial to determine if the XPC-mediated reduction demonstrated in the first trial changed over time. As characteristic of this specific *in vitro* model (data not shown), there is a bloom of *Campylobacter* from 0 to 24 h (Figure 2B). This is likely due to the abundance of nutrients available in Bolton’s broth and the ceca. There was an effect of treatment (XPC vs. CON) and an effect of time (*P* < 0.001) and there was a trend toward the interaction of time and treatment being important (*P* = 0.063). Using Dunnet’s test setting 0 h as the comparison, 24 h and 48 h were significantly different than 0 h (*P* < 0.001; Figure 2B). Additionally, the main effect of treatment was also significant (*P* = 0.0124) when comparing the CON group to the XPC group (Figure 2). The lack of numerical differences between 24 and 48 h in the XPC + *C. jejuni* group is likely driving the lack of significance in the interaction when compared to the *C. jejuni* + 0.1% ceca alone (*P* = 0.063).

Regarding the effect of treatment, there was a significant reduction in log cells/mL of *C. jejuni* recovered in XPC treatment groups vs. CON (*P* = 0.0124). When separating out the means using Dunnet’s multiple comparisons setting CON as the contrast, there is a sustained reduction in *C. jejuni* in XPC as compared to the CON group. Therefore, the stability of *C. jejuni* populations in both groups are numerically different from one another, though lack statistical significance as the rate of change is not different from 24 to 48 h.

### Microbiome Analysis: Alpha and Beta Diversity Analyses

Alpha and beta diversity analysis is an essential first step in understanding how the XPC impacts the microbiome, and, in turn, is mechanistically tied to the reduction of *Campylobacter*. Alpha diversity focuses on the richness of the sample, which is defined as unique operational taxonomical units [OTU(s)] per sample as well as how even that count is between samples or treatment groups. Whereas beta diversity focuses on the dissimilarity of OTUs between samples or treatment groups with or without phylogenetic alignments playing a factor.

**FIGURE 3:**
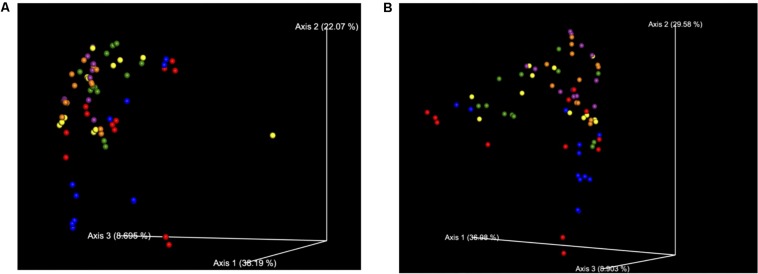
**(A)** Bray Curtis Diversity Plot. The PCoA of the Bray–Curtis Diversity Plot. Key: Red: 0 h; CON: Blue; 0 h XPC: Orange: 24 h CON; Green: 24 h XPC; Purple: 48 h CON; Yellow 48 h XPC. **(B)** Weighted Unifrac Diversity Plot. The PCoA of the Weighted Unifrac Diversity Plot. Key: Red: 0 h; CON: Blue; 0 h XPC: Orange: 24 h CON; Green: 24 h XPC; Purple: 48 h CON; Yellow 48 h XPC.

All available indices for alpha diversity were conducted in QIIME2. The alpha diversity results are shown in Table 1. Pielou’s Evenness and Shannon Diversity indices were statistically significant throughout the course of the study (*P* < 0.05). Pielou’s evenness speaks to how consistent the unique OTUs were between samples, whereas Shannon’s Diversity focuses on the number of unique samples per sample. Together, both indices provide a complete description of the alpha diversity in this study. Differences between time and treatment were observed with both metrics (Pileou Evenness; Figure 3A, Shannon Diversity Index; Figure 3B and Table 1). There was no significant difference in the alpha diversity using either indices at 0 h between XPC and CON. There were significant differences in diversity over time as compared to 0 h, with an increase in the *H*-score in control and CON independently over time. This indicated that time drove many of the differences observed, which parallels the *C. jejuni* microbiology data. When comparing treatments, there were no significant differences at the same time points. However, there were significant differences in alpha diversity as compared to 0 h for all treatment groups. Therefore, the number of unique OTUs and the evenness between samples is not significantly different over comparable time points by treatment. Any differences in the microbiota are due to microbial compositional diversity, not individual OTU count.

**TABLE 1:** Changes in Shannon Diversity Index and Pilou’s Evenness.

**Kruskal–Wallis pairwise comparisions**				**Shannon Diversity Index**			**Evenness Index**		
**Group**	***n***	**Group**	***n***	***H***	***P*-value**	***Q*-value**	***H***	***P*-value**	***Q*-value**
0 h CON	(*n* = 12)	0 h CON	(*n* = 12)	1.92	0.17	0.25	2.43	0.12	0.15
**0 h CON**	**(*n* = 12)**	**24 h CON**	**(*n* = 12)**	**5.88**	**0.02**	**0.04**	**8.0033**	**0.00**	**0.02**
0 h CON	(*n* = 12)	24 h XPC	(*n* = 12)	4.56	0.03	0.06	3.8533	0.05	0.07
**0 h CON**	**(*n* = 12)**	**48 h CON**	**(*n* = 12)**	**7.05**	**0.01**	**0.04**	**9.0133**	**0.00**	**0.01**
**0 h CON**	**(*n* = 12)**	**48 h CON**	**(*n* = 12)**	**5.33**	**0.02**	**0.04**	3.8533	0.05	0.07
**0 h XPC**	**(*n* = 12)**	**24 h CON**	**(*n* = 12)**	**7.68**	**0.01**	**0.04**	**10.083**	**0.00**	**0.01**
**0 h XPC**	**(*n* = 12)**	**24 h XPC**	**(*n* = 12)**	**6.16**	**0.01**	**0.04**	**6.1633**	**0.01**	**0.04**
**0 h XPC**	**(*n* = 12)**	**48 h CON**	**(*n* = 12)**	**8.67**	**0.00**	**0.04**	**10.083**	**0.00**	**0.01**
**0 h XPC**	**(*n* = 12)**	**48 h XPC**	**(*n* = 12)**	**6.45**	**0.01**	**0.04**	**5.88**	**0.02**	**0.04**
24 h CON	(*n* = 12)	24 h XPC	(*n* = 12)	0.96	0.33	0.44	3	0.08	0.11
24 h CON	(*n* = 12)	48 h CON	(*n* = 12)	0.56	0.45	0.52	0.6533	0.42	0.45
24 h CON	(*n* = 12)	48 h XPC	(*n* = 12)	0.48	0.49	0.52	2.2533	0.13	0.15
24 h XPC	(*n* = 12)	48 h CON	(*n* = 12)	2.25	0.13	0.22	4.32	0.04	0.07
24 h XPC	(*n* = 12)	48 h XPC	(*n* = 12)	0.05	0.82	0.82	0.2133	0.64	0.64
24 h XPC	(*n* = 12)	48 h XPC	(*n* = 12)	0.85	0.36	0.44	4.32	0.04	0.07

**Evenness**									
**Group**	**1**	**Group**	**2**						

0exp3Sham	(*n* = 12)	0exp3XPC	(*n* = 12)						
**0exp3Sham**	**(*n* = 12)**	**24exp3Sham**	**(*n* = 12)**						
0exp3Sham	(*n* = 12)	24exp3XPC	(*n* = 12)						
**0exp3Sham**	**(*n* = 12)**	**48exp3Sham**	**(*n* = 12)**						
0exp3Sham	(*n* = 12)	48exp3XPC	(*n* = 12)						
**0exp3XPC**	**(*n* = 12)**	**24exp3Sham**	**(*n* = 12)**						
**0exp3XPC**	**(*n* = 12)**	**24exp3XPC**	**(*n* = 12)**						
**0exp3XPC**	**(*n* = 12)**	**48exp3Sham**	**(*n* = 12)**						
**0exp3XPC**	**(*n*** = **12)**	**48exp3XPC**	**(*n* = 12)**						
24exp3Sham	(*n* = 12)	24exp3XPC	(*n* = 12)						
24exp3Sham	(*n* = 12)	48exp3Sham	(*n* = 12)						
24exp3Sham	(*n* = 12)	48exp3XPC	(*n* = 12)						
24exp3XPC	(*n* = 12)	48exp3Sham	(*n* = 12)						
24exp3XPC	(*n* = 12)	48exp3XPC	(*n* = 12)						
48exp3Sham	(*n* = 12)	48exp3XPC	(*n* = 12)						

*The significant pair-wise differences as determined by Kruskal–Wallis Pairwise Comparisons are bolded, with a *Q* < 0.05 being considered significant.*

**FIGURE 4:**
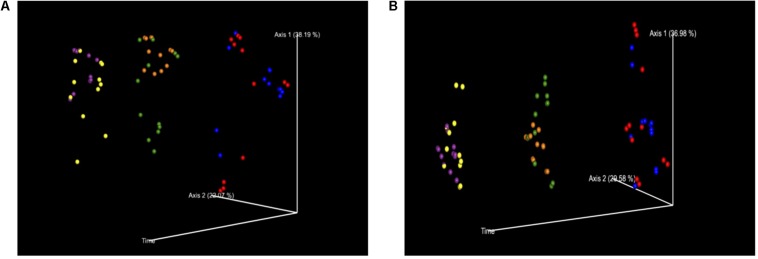
**(A)** PCoA of Bray–Curtis Diversity Index separated out by time. Time was a significant factor in the change in diversification and therefore the treatment groups were plotted against the variable of time. Key: Red: 0 h; CON: Blue; 0 h XPC: Orange: 24 h CON; Green: 24 h XPC; Purple: 48 h CON; Yellow 48 h XPC. **(B)** PCoA of Weighted Unifrac Diversity Index Separated Out By Time. Time was a significant factor in the change in diversification and therefore the treatment groups were plotted against the variable of time. Key: Red: 0 h; CON: Blue; 0 h XPC: Orange: 24 h CON; Green: 24 h XPC; Purple: 48 h CON; Yellow 48 h XPC.

However, unlike the alpha diversity analyses, beta diversity began to exhibit significant differences between groups at specific time points. All of the available analytics were conducted when evaluating whether or not specific metrics for beta diversity were statistically important. Both Bray–Curtis (Figure 3A), and the weighted unifrac distance matrix (Figure 3B) indices, were statistically significant between treatments (*P* < 0.05; Table 2). The plots are visualized in Figures 4A,B, and the statistical analysis indicated profound differences in beta diversity.

**TABLE 2:** Changes in Bray–Curtis and Weighted Unifrac Diversity Indices.

**ANISOM comparisons**				**Bray–Curtis Diversity Index**			**Weighted Unifrac Diversity Index**		
**Group**	**Group**	**Sample**	**Permutations**	**pseudo-*F***	***P*-value**	***Q*-value**	**pseudo-*F***	***P*-value**	***Q*-value**
0 h CON	0 h XPC	24	999	1.73	0.18	0.21	1.40	0.23	0.27
0 h CON	24 h CON	**24**	**999**	**6.31**	**0.00**	**0.01**	**6.68**	**0.00**	**0.00**
0 h CON	24 h XPC	24	999	1.75	0.15	0.19	1.97	0.14	0.18
0 h CON	48 h CON	**24**	**999**	**6.36**	**0.00**	**0.01**	**7.13**	**0.00**	**0.01**
0 h CON	48 h XPC	24	999	2.08	0.11	0.15	2.33	0.07	0.10
0 h XPC	24 h CON	**24**	**999**	**9.96**	**0.00**	**0.01**	**11.02**	**0.00**	**0.00**
0 h XPC	24 h XPC	**24**	**999**	**4.90**	**0.00**	**0.01**	**5.04**	**0.01**	**0.01**
0 h XPC	48 h CON	**24**	**999**	**10.55**	**0.00**	**0.01**	**12.29**	**0.00**	**0.00**
0 h XPC	48 h XPC	**24**	**999**	**3.79**	**0.01**	**0.01**	**4.22**	**0.00**	**0.00**
24 h CON	24 h XPC	**24**	**999**	**6.36**	**0.00**	**0.01**	**4.88**	**0.01**	**0.01**
24 h CON	48 h CON	24	999	0.32	0.85	0.85	0.42	0.69	0.69
24 h CON	48 h XPC	24	999	2.23	0.01	0.01	2.42	0.03	0.05
24 h XPC	48 h CON	**24**	**999**	**5.56**	**0.01**	**0.02**	**4.49**	**0.02**	**0.03**
24 h XPC	48 h XPC	24	999	1.02	0.35	0.38	0.68	0.56	0.60
48 h CON	48 h XPC	**24**	**999**	**2.10**	**0.02**	**0.03**	2.40	0.04	0.06

**Weighted**									
**Group**	**1**	**Sample**	**Permutations**						

0exp3Sham	0exp3XPC	24	999						
0exp3Sham	24exp3Sham	24	999						
0exp3Sham	24exp3XPC	24	999						
0exp3Sham	48exp3Sham	24	999						
0exp3Sham	48exp3XPC	24	999						
0exp3XPC	24exp3Sham	24	999						
0exp3XPC	24exp3XPC	24	999						
0exp3XPC	48exp3Sham	24	999						
0exp3XPC	48exp3XPC	24	999						
24exp3Sham	24exp3XPC	24	999						
24exp3Sham	48exp3Sham	24	999						
24exp3Sham	48exp3XPC	24	999						
24exp3XPC	48exp3Sham	24	999						
24exp3XPC	48exp3XPC	24	999						
48exp3Sham	48exp3XPC	24	999						

*The relationships that are significantly different from one another as determined by ANISOM are bolded. A *Q* < 0.05 is considered significant.*

Bray–Curtis (BC) Beta Diversity evaluates the quantitative dissimilarity of OTUs between samples. There was no difference in BC at 0 h when directly comparing XPC vs. the CON. There was also no difference between 0 h CON and XPC. However, there are statistically significant differences when comparing CON at 0 h vs. 24 and 48 h. Conversely, there is no difference in BC diversity between CON at 24 and 48 h, indicating that the fluctuations in beta diversity occurred within the control group after 24 h. When comparing CON vs. XPC at 24 h, there was a statistically significant change in beta diversity between the groups (*Q* = 0.0075). At 48 h, there were significant changes in beta diversity between XPC and the control group (*Q* = 0.03). When comparing beta diversity across time in the XPC treatment group, there were significant changes in diversity from 0 h and 24 and 48 h. There was no significant change in BC diversity between 24 and 48 h in the XPC treated groups.

Weighed Unifrac (WU) Beta Diversity Matrices quantitatively measures dissimilarity between samples and considers the phylogenetic relationships. There was no difference in WU diversity between CON and XPC at 0 h. Therefore, any differences occurring thereafter were due to time and treatment. Comparing the CON group across all time points, there were differences at 0 vs. 24 h, and 0 vs. 48 h. When comparing XPC and CON at the same time points, there were significant differences in WU diversity at 24 h but not 48 h. Finally, differences in WU diversity in XPC groups was significantly different at 0 vs. 24 h and 0 and 48 h.

In order to fully visualize whether or not the effect of treatment was due to the continuous variable of time, principle coordinate analysis (PCoA) plots were constructed to evaluate the contribution that time contributed to the data variability between treatment groups for both BC (Figure 4A) and WU (Figure 4B) analyses. The further down the axis the data point was, the more that axis contributed to the variability of the data. The greater the distance down the axis there was between groups, the greater the effect of time had on the microbiome. It is clearly demonstrated in both plots that when defining time as a contributing variable, significant differences in beta diversity demonstrated in Table 2 were readily visualized. Therefore, there were significant divergences between different treatment groups, which is not only driven by treatment, but is also heavily influenced by time, specifically 0–24 h.

### Microbiome Analysis: Taxonomy Visualization and ANCOM Analysis of Compositional Diversity Differences

**FIGURE 5:**
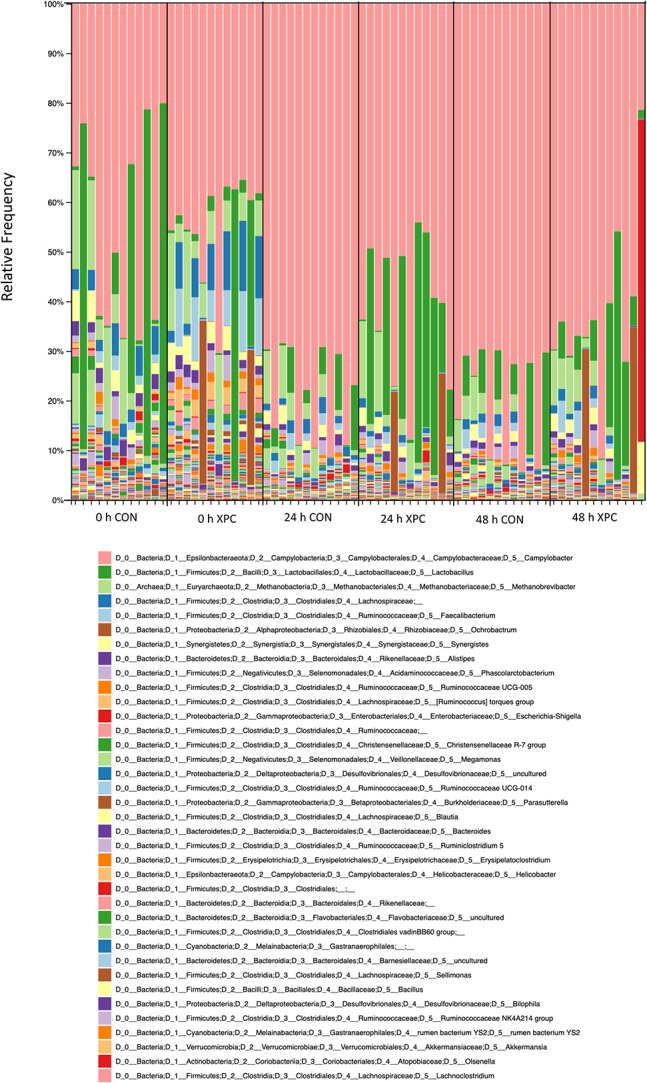
Taxonomical bar graphs. Taxonomy bar plots are visualized through the species epithet and the colors are presented from the top of the graph (coral, *Campylobacter*) to the bottom of the graph (coral, *Lachnoclostridium*).

**FIGURE 6:**
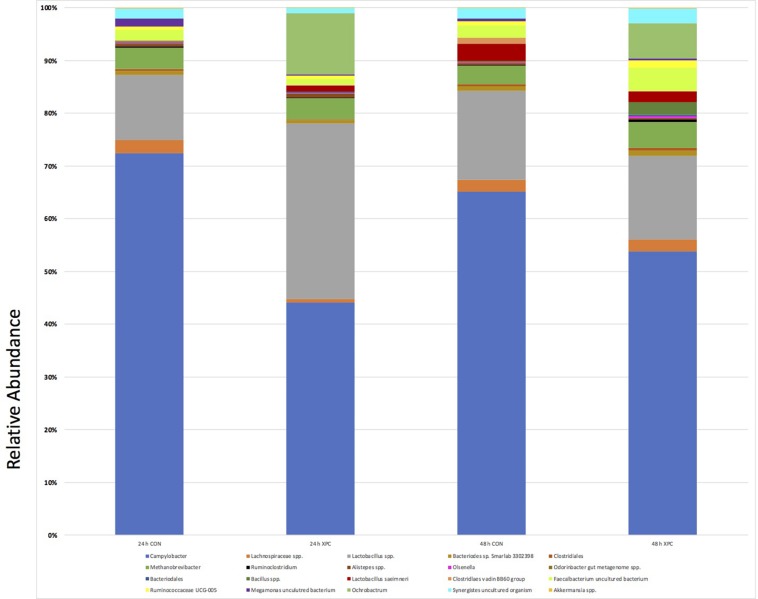
ANCOM Analysis of Treatment Groups 24 and 48 h. The most prevalent groups that were identified by ANCOM as statistically significant are presented. Differences between the groups were identified as significant by ANCOM (*Q* < 0.05).

After the alignment, taxonomical bar plots were created to give a complete visualization of the microbial diversity as supported by ANCOM analysis (species epithet; Figure 5). The declaration of compositional differences cannot be accomplished accurately by comparing individual OTU counts between group, nor can trends in diversity as the qualitative approach lacks the incorporation of an accurate false discovery rate (Figure 6). As beta diversity analysis demonstrated compositional divergence between groups, ANCOM was conducted at all taxonomical levels (data not shown) to evaluate which microorganisms are unique. ANCOM was chosen due to its ability to increase statistical power with small datasets while effectively incorporating a stringent false discovery rate (Mandal et al., 2015). The use of a false discovery rate of appropriate vigor is important for multi-dimensional, multi-response data characteristic of a single sample analyzed via next-generation sequencing.

Stark differences between XPC and CON groups at 24 and 48 h were observed at L7 (*species* epithet; Figure 6). Data was sorted to visualize the top nine species, though numerous species were present and exported into Excel. Importantly, the OTUs mapped to *Campylobacter* (light blue) paralleled the microbiological data. Therefore, the XPC mediated reduction in *C. jejuni* is supported by Petroff-Hausser counting, microscopy, and sequencing. The next highest abundant group is *Lactobacillus* spp. (gray), which was significantly enriched in the XPC groups as compared to the control groups. The OTUs that mapped to *Lactobacillus* spp. were over 3.8-fold more abundant at 24 h than XPC treated groups vs. CON. The next group with the greatest significant difference between groups was *Methanobrevibacter* spp. There were about 1.4-fold more *Methanobrevibacter* spp. in XPC treated groups, than the CON group. *Lachnospiraceae* spp. (orange) are differentially abundant between groups, however, unlike previously abundant species, *Lachnospiraceae* was reduced 7.9-fold at 24 h in XPC treated groups, then increased eightfold at 48 h. *Lachnospiraceae* at 48 h is slightly higher than CON groups. Similarly, there is a contraction, followed by a bloom of an OTU identified as an uncultured Firmicutes termed *Phascolarctobacterium* spp. *Faecalbacterium* shares a similar trend, with a significant bloom in the population by 48 h. Two other groups that were enriched *Synergistes* and *Bacteroides* sp. Smarlab 3302398 shared similar trends. Finally, *Megamonas* spp., or formerly *Bacteriodes hypermegas*, showed a different trend, contracting significantly between CON and XPC groups at 24 h, then remaining contracted throughout the rest of the study. XPC groups had less *Megamonas* spp. present than other groups. Therefore, multiple species of bacteria are differentially expressed in XPC vs. the CON group, though their specific role remains to be elucidated.

## Discussion

Modulating the microbiome has the potential to control *Campylobacter* infections since the cecal microbiota and *Campylobacter* appear to be extensively interconnected (Indikova et al., 2015). This study was an *in vitro* approach for evaluating whether XPC could modulate the microbiome and if those effects could reduce *C. jejuni* independent of the immune system. Results suggest that XPC-mediated reductions of *Campylobacter* are cecal dependent, which is likely driven by changes in GIT microbial ecology, which is consistent with previously published reports for *Salmonella* (Rubinelli et al., 2016; Park et al., 2017). When the experiment was repeated and extended through 48 h, there was no increase in *C. jejuni* load and the effect of XPC was sustained as compared to the CON group. Incorporating the Petroff-Hausser counter was important in this study as it detected viable-but-not-culturable *C. jejuni*, which can result in foodborne disease and are not detected using traditional microbiological methods (Ziprin et al., 2003). However, direct evidence associated with the infectivity of viable-but-not-culturable bacteria as infectious agents is not strong (Ziprin et al., 2003).

Evidence that the microbiome can be harnessed to reduce *C. jejuni* fitness in the poultry GIT is twofold: first, *C. jejuni* colonization is influenced by total microbiome maturation and diversity; second, there are populations of bacteria that are directly antagonistic to *C. jejuni* (Awad et al., 2016; Connerton et al., 2018). In order for a feed additive to be successful, we theorize that it would likely have to exhibit a multi-dimensional influence on the microbial ecology of the gut to be sustainable enough to reduce *Campylobacter* colonization of poultry. The microbiome data independent of the immune system indicates that XPC seems to produce specific changes to the microbiome of the poultry ceca as demonstrated in this *in vitro* model. Importantly, there is not just one prokaryote population that seems to be driving these effects. In fact, ANCOM analysis indicates that the XPC-mediated changes to the microbiome are dynamic and potentially specific to XPC. For a pathogen such as *Campylobacter* that exhibits genomic and metabolic plasticity, total compositional changes to the microbiome may be essential in mitigating the threat of the pathogen. This could be why more one-dimensional approaches, such as probiotics that are transient, are not completely effective. For example, when Thibodeau et al. (2015) fed a selenium-yeast probiotic product traditionally tied to improved immunobiology and feed efficiency, the researchers did not detect microbiota, *Campylobacter* or general bird health responses in experimentally inoculated birds. *Lactobacillus* and other probiotics exhibit similar ranges in efficacy, which may be due, in part, to their ability to modulate the microbiome (Broom and Kogut, 2018).

However, additional evidence suggests that XPC is more beneficial to the bird, with microbiome-activating effects. Guyard-Nicodème et al. (2016) tested a number of commercial products, including XPC, as feed additives administered throughout the grow-out period to control experimentally infected *Campylobacter*. They found XPC significantly reduced a late *C. jejuni* challenge, but not early, in the grow-out period. Considering the *in vitro* data and other evidence indicate the importance of a window of opportunity driven by the maturity index of the microbiome, it may be interesting to see if XPC changes the rate of diversification, maturity, and stability of the microbiome. Altogether, such data could drive the effects demonstrated by Guyard-Nicodème et al. (2016).

The identification of specific bacterial species, or cornucopia of bacterial species, as biomarkers associated with *Campylobacter* infection and resistance has been studied. Evidence suggests that microbiota natively and significantly enriched with *Bacteriodes* and *Escherichia* increased the likelihood of native infections of *Campylobacter* in chicken abattoir workers (Dicksved et al., 2014). However, a microbiota of human subjects prior to infection enriched with *Lachnospiraceae*, *Clostridiales*, and *Anaerovorax* conferred a general resistance to *Campylobacter* and resulted in less volatility over time (Dicksved et al., 2014). *Lachnospiraceae* and *Clostridiales* were the most differentially expressed species between treatments and across time throughout this study. More studies are needed to validate this phenotypically and if the microbiome identified in humans that confers robustness to *Campylobacter* assault is paralleled in the broiler chicken.

Poultry associated lactic acid bacteria have been previously demonstrated to inhibit the growth and repress virulence properties of *Campylobacter*, which is why *Lactobacillus* probiotics are popular in the poultry industry (Stern et al., 2005; Santini et al., 2010). Moreover, several studies have identified bacteriocins as the mechanism behind this effect. Bacteriocins like nisin and SMXD51 and NRRL B-30514, of *Lactobacillus salivarius*, kill *C. jejuni* and *Campylobacter coli* as well as several other bacterial species (Stern et al., 2005; Messaoudi et al., 2012). This same strain of *L. salivarius* also reduced a *C. jejuni* challenge in broilers when repeatedly administered orally (Stern et al., 2005; Saint-Cyr et al., 2017). Another potentially antagonistic species was *Megamonas* spp. Some evidence suggests that *Megamonas* may be antagonistic to *Campylobacter* persistence in the poultry GIT (Scumpham et al., 2010).

*Faecalbacterium* is a significant anaerobic butyrate metabolizer and signifies a healthy gut axis. *Faecalbacterium*, along with *Lactobacillus*, is positively associated with high performing broiler chickens as well as beneficially modulating the innate immune system (Yan et al., 2017). *Faecalbacterium* is also being investigated as a probiotic as increasing the populations of *Faecalbacterium* improve immune function, overall physiological functioning, and help fortify the microbiome against inflammatory assault (Sokol et al., 2008; Yan et al., 2017). *Faecalbacterium* is also associated with high microbial diversity (Sokol et al., 2008). It is theorized that *Faecalbacterium* increases the microbiome robustness to disbyosis by inducing IL-10 and other anti-inflammatory cytokines, as well as increasing butyrate production Sokol et al., 2018). As XPC in feed results in increased butyrate and other short chain fatty production, it is interesting that many of the differentially abundant bacteria are also high butyrate producers.

Data also suggests that volatility of that microbiota over time may predispose certain populations to Campylobacteriosis more so than the enrichment or loss of certain microbial populations (Dicksved et al., 2014). Those differences in alpha and beta diversity are likely consistent in susceptible populations. In poultry, *C. jejuni* thrives when the microbiome is its most volatile, which is prior to immunobiological and microbiome maturity (Connerton et al., 2018). Therefore, increasing the rate of maturation and the stability of the microbiome with in-feed additives may be a beneficial avenue for producers. The stabilization of the microbiome may be indicated as a potential mechanism for XPC efficacy via the differential abundance of *Methanobrevibacter* spp., which is a methanogen producing archaeon associated with the digestion of complex carbohydrates by being a major consumer of bacterial fermentation byproducts such as hydrogen and carbon dioxide (Armougom et al., 2009). Methanogens can be detected in the fecal material relatively early during a broiler life cycle and *Methanobrevibacter woesii* has been identified as the predominant methanogen in adult layer hen ceca (Saengkerdsub et al., 2007a, b). Additionally, *Methanobrevibacter* spp. and other methanogens are important in a gut rich in anaerobic fermentation as the accumulation of H_2_ can be deleterious to the host (Gill et al., 2006). When microbial populations are healthy, anaerobic, and actively producing short chain fatty acids, *Methanobrevibacter* are usually enriched (Gill et al., 2006). This is consistent with detectable methane production occurring in poultry cecal *in vitro* anaerobic cultures grown on either a high fiber diet or a grain-based layer ration (Saengkerdsub et al., 2006).

Another important organism, *Phascolarctobacterium* spp., which produces acetate and propionate is also present and differentially abundant in this study, and are also considered indicators of an efficient metabolic state (Wu et al., 2017). *Synergistetes* are obligate anaerobic bacterial species that are amino acid reducers, although not much is known about the species beyond anecdotal knowledge (Jumas-Bilak et al., 2009). However, it is a known species associated with a “normal” microbiome. Therefore, an increase in anaerobic populations associated with a stable, energetically balanced microbiome, while also producing a microbiota that is competitively exclusive and antagonistic to *Campylobacter*, may be a mechanism for XPC anti-*Campylobacter* efficacy.

Significant caveats exist with the proposed *in vitro* model. First, short of oscillation, we do not simulate peristalsis or continuous culture environments. This likely reduces the mechanical stress on the microbiota as well as limits nutrient availability over time and greatly limits the potential applications of this model. We overcome the continuous culture limitation by only allowing the culture to go out to 48 h. Additionally, the effect of time was not likely driving the treatment effects as presented by our data. However, the authors agree that additional validations will be required, specifically the comparison of this model to a live bird study, to evaluate the specific caveats associated with time, nutrient availability, and antibiotic components of the media. Second, the difficulties in recovering *Campylobacter* were overcome by using the standard Bolton’s Broth preparations. This absolutely can change the potential treatment-based fluctuations of the microbial ecology of the *in vitro* model and bias the data. Antibiotic resistance is favored with certain populations. Therefore, it is reasonable to believe that there may be important populations that may either benefit or antagonize *Campylobacter* that are not present in this study. Therefore, a full validation of this model will be required moving forward that tunes the trade-off between *Campylobacter* recovery and background microbiota. However, what is interesting about this study is that changes to the microbiota take place in the presence of XPC and ultimately XPC treatment groups carry fewer *Campylobacter*. This is mirrored in the cell counts as well as in the sequencing data. While caveated, with the controls in place, this study provides, preliminary understanding of the effects of XPC.

While single approaches, like a probiotic, have important effects, multiple antagonistic species were identified, potentially indicating that XPC effects may be multi-modal. This study provides preliminary evidence to suggest that XPC may ultimately change the ecology of the gut and be exclusive to not only *Salmonella*, but *Campylobacter* as well. Overall, it appears that there is an overall environmental shift from CON to XPC groups that are sustained over time that promote anaerobic production and oxidation of fatty acids and complex carbohydrates by Archeae and prokaryotes. Even if the population of bacteria or Archeae are reduced initially, they rebound by 48 h, which may indicate that XPC continues to beneficially modulate the microbiome over time. Additionally, bacteria that actively resist *Campylobacter*, such as *Lactobacillus* spp., were also identified in this study. Therefore, the total microbial shift in the environment may contribute to the reduction of *Campylobacter*. Further analyses evaluating metabolomics changes to the microbiome must also be conducted to determine if the phenotypic changes of the gut match the species identified through 16S microbiome sequencing.

## Conclusion

A previous report estimated that reducing *Campylobacter* in the chicken cecum by as little as three logs could reduce the risk of human disease by as much as 90–100% (Romero-Barrios et al., 2013). While the reductions in *Campylobacter* are not that biologically significant in this current study, it is promising. By understanding how *Campylobacter* persists, and the microbiota ecology associated with resistance and infection, new management strategies may be developed. Therefore, a necessary next step is an experimental infection of *Campylobacter jejuni* in broiler chickens to monitor birds for anti-*Campylobacter* effects. Necessarily, evidence presented in this paper highly suggests such data should include temporal studies in microbial diversity, maturation, and volatility. Overall, this study also suggests that XPC requires the microbiota for anti-pathogenic effects, which results in profound changes to the microbiome that both competitively exclude *Campylobacter* and may be directly antagonistic to the pathogen. By increasing butyrate producing bacteria, phenotypic data evaluating XPC-mediated metabolite changes in the microbiota from previous studies is further supported (Possemiers et al., 2013; Rubinelli et al., 2016).

Increases in butyrate producing bacteria are both anti-pathogenic and immune-balancing. Data presented herein supports other publications that evaluate the effects of XPC in poultry or the microbiome, where butyrate production was noted (Possemiers et al., 2013; Feye et al., 2016; Rubinelli et al., 2016). By fortifying the immunobiological-microbiome axis through the production of metabolites like butyrate, overall changes to host pathology are possible. Therefore, the underlying mechanism that drives the overall effects of XPC may rest at that nexus of XPC, the microbiome, and the immune system. Future studies need to elucidate that nexus, which could profoundly enhance the utility of XPC to producers.

## Data Availability Statement

The authors submitted the raw, unprocessed, and demultiplexed figures onto the laboratory GitHub site, along with the necessary metadata file. The repository is freely accessible and available at: https://github.com/RickeLab/FrontiersMicroManID493430.

## Ethics Statement

Ethical review and approval was not required for the animal study because ceca were collected in a commercial plant, no need for IACUC approval as the animals were processed as per industry standards.

## Author Contributions

PR, SR, HP, and MK conceived the experiment. KF executed the microbiome analytics, developed the bioinformatics pipeline, conducted the microbiome analysis and interpretation, analyzed the data, prepared the figures, wrote the manuscript, and handled submissions and edits. PR conducted the *in vitro* work, executed the sequencing run, processed the samples, prepared the first figure, and edited the manuscript. KF, SR, HP, PR, and WC edited the final manuscript. WC provided significant insight to the analytics. All authors were given the opportunity to edit the manuscript prior to its final submission.

## Conflict of Interest

HP and WC are employees of Diamond V.

The remaining authors declare that the research was conducted in the absence of any commercial or financial relationships that could be construed as a potential conflict of interest.

## Footnotes

1 www.basespace.illumina.com
